# A Simple and Label‐Free Gold Nanoparticle Assay and Duplex PCR for Rapid Detection of *Klebsiella pneumoniae* K2

**DOI:** 10.1002/mbo3.70281

**Published:** 2026-03-30

**Authors:** Sepideh Ahmadi, Fatemeh Haddadi, Hossein Kamaladini

**Affiliations:** ^1^ Department of Biology Faculty of Sciences University of Zabol Zabol Iran; ^2^ Department of Medical Biotechnology, School of Advanced Technologies in Medicine, Student Research Committee Shahid Beheshti University of Medical Sciences Tehran Iran

**Keywords:** colorimetric assay, duplex PCR, gold nanoparticles (AuNPs), K2A and orf10 genes, Klebsiella pneumoniae, nanobiosensor

## Abstract

Rapid, accurate, and cost‐effective diagnostic tools are crucial for the early detection of bacterial pathogens, particularly *Klebsiella pneumoniae*, a leading cause of multidrug‐resistant infections. In this study, we developed two molecular detection approaches: a simplified, label‐free colorimetric assay using unmodified gold nanoparticles (AuNPs) with direct DNA addition, and a duplex PCR assay targeting the *K2A* and *orf10* genes, two key virulence and identification markers of *K. pneumoniae*. The unmodified AuNP assay, employing a 20 bp K2A gene probe, enabled visible color change detection within 60 min, without the need for complex instrumentation, achieving a sensitivity of 1.56 ng/μL. Duplex PCR successfully amplified fragments of 532 bp (K2A) and 309 bp (orf10), with high sensitivity levels of 1 pg/μL and 0.07 ng/μL, respectively. This integrated strategy offers both visual rapid screening and molecular confirmation in a single workflow, improving diagnostic reliability while maintaining low cost and simplicity. The use of unmodified AuNPs eliminates the need for chemical functionalization, significantly reducing assay preparation time and cost. Together, these features make the proposed method a promising platform for point‐of‐care molecular diagnostics and epidemiological monitoring of *K. pneumoniae* in resource‐limited settings.

## Graphical abstract

A rapid, cost‐effective dual‐detection strategy for Klebsiella pneumoniae K2 was developed by combining a label‐free AuNP colorimetric assay (LOD: 1.56 ng/μL, 60 min) with duplex PCR targeting K2A and orf10 genes (LOD: 1 pg/μL). This approach enables rapid screening with accurate genetic validation for point‐of‐care diagnostics in resource‐limited settings.

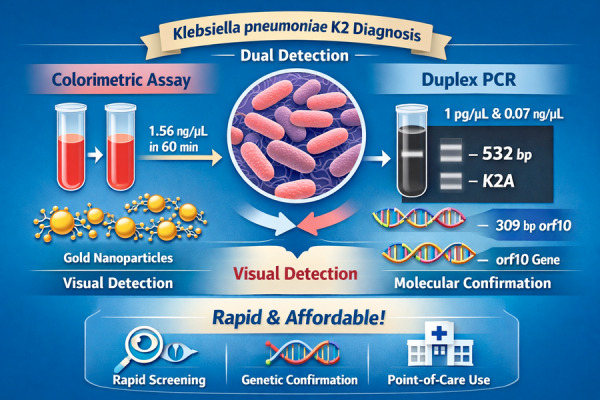

## 1 Introduction

The identification of pathogenic bacteria using traditional diagnostic techniques based on phenotypic characteristics typically requires several days, delaying clinical decision‐making and treatment initiation (Lauri and Mariani 2009). In contrast, molecular techniques have revolutionized pathogen detection by offering rapid, sensitive, and specific analysis of microbial DNA. Over the past two decades, significant advances in PCR technology and other nucleic acid amplification techniques have established molecular diagnostics as indispensable tools in clinical microbiology (Khan 2014; Bej et al. 1991). Among them, multiplex and duplex PCR have expanded analytical capacity by enabling simultaneous amplification of multiple target sequences, thereby improving efficiency and diagnostic precision in culture‐independent detection of infectious agents (Aydemir et al. 2014; Anbazhagan et al. 2010; Li et al. 2012).

Recent breakthroughs in nanotechnology have further accelerated the development of highly sensitive and rapid diagnostic tools. The integration of nanomaterials in biosensor platforms has given rise to nanobiosensors, which combine molecular recognition with nanoscale signal transduction, resulting in enhanced sensitivity and shorter detection times (Prasad 2014). Among nanomaterials, gold nanoparticles (AuNPs) have attracted particular attention due to their remarkable optical, electronic, and physicochemical properties, including high biocompatibility, stability, non‐toxicity, and controllable morphology (Jain et al. 2012; Abdulkin et al. 2014; Mieszawska et al. 2013). Their strong surface plasmon resonance effect enables the development of colorimetric assays that translate molecular interactions into visible color changes. This approach has been successfully applied for the rapid, low‐cost detection of various pathogens, biomolecules, and gene expression events (Ghazi et al. 2018; Ahmadi et al. 2018), offering a powerful alternative to conventional detection systems.

The principle of AuNP‐based colorimetric detection relies on the aggregation state of nanoparticles in response to nucleic acid hybridization. In the presence of target DNA, complementary hybridization between the probe and target sequence stabilizes the nanoparticles, maintaining the red color of the solution. In the absence of target DNA, single‐stranded probes adsorb onto the AuNPs, stabilizing them and preventing aggregation. However, when NaCl is added, unprotected AuNPs aggregate, causing a visible color shift from red to purple. The optical change can be easily monitored by the naked eye or spectrophotometric methods, providing a label‐free, equipment‐free, and cost‐efficient diagnostic platform.

Optimization of key reaction components including probe, salt, and buffer concentrations is crucial for maintaining the accuracy of colorimetric assays. Excess probe concentration may lead to nonspecific interactions with both the target DNA and the AuNPs, resulting in no visible color change. Similarly, excessively high salt concentrations can neutralize surface charges on AuNPs, destabilizing the colloidal system and causing false‐positive aggregation.

*Klebsiella pneumoniae* is a Gram‐negative, non‐motile, rod‐shaped bacterium belonging to the Enterobacteriaceae family and is a major cause of hospital‐acquired infections (Wu et al. 2021). It is among the top eight nosocomial pathogens worldwide (Chen et al. 2012), responsible for diverse infections including pneumonia, meningitis, urinary tract infections, and wound infections (Hu et al. 2020). Of particular concern is its association with pyogenic liver abscesses (PLA), a life‐threatening condition increasingly reported in Asia (Oikonomou and Aye 2017; Hsu et al. 2016), the United States (Rahimian et al. 2004), and Europe (Sobirk et al. 2010). Among the pathogenic serotypes, K2 is recognized as one of the most virulent and frequently associated with PLA (Siu et al. 2012).

Capsular polysaccharide synthesis (CPS) plays a central role in *K. pneumoniae* virulence. The K2A (orf9) gene is a serotype‐specific marker responsible for capsule formation, while the orf10 gene is involved in capsule translocation and virulence. These conserved and serotype‐specific genetic regions represent reliable molecular targets for diagnostic assay development. With the growing availability of genome sequences in public databases such as GenBank, the identification of such molecular signatures has greatly facilitated the design of precise, gene‐based diagnostic assays for clinical pathogens.

In our previous work, we developed a colorimetric assay using thiolated DNA probes for bacterial detection (Mousivand et al. 2023). While that study demonstrated the high sensitivity of gold nanoprobe‐based detection systems, probe modification adds cost, complexity, and time to assay preparation. To address these challenges, the present study introduces a simplified, label‐free colorimetric assay employing unmodified AuNPs for detecting *K. pneumoniae*, and compares its performance with a duplex PCR targeting the *K2A* and *orf10* genes. Similar advancements have been reported in recent literature, where AuNP‐based biosensors provided rapid and highly sensitive detection of bacterial and viral pathogens (Parkhe and Tiwari 2024; Celik et al. 2023; Bagherinajafabad et al. 2023; Khoshraftar et al. 2024; Mayaka and Alocilja 2025; Saikaew et al. 2025). These studies highlight the growing importance of nanoparticle‐based molecular diagnostics as powerful, low‐cost, and field‐deployable tools, especially for resource‐limited settings.

Therefore, this study aimed to design and evaluate a dual molecular diagnostic strategy, a colorimetric assay using unmodified AuNPs and a duplex PCR system for the specific detection of *K. pneumoniae* through its capsular polysaccharide biosynthesis genes. This work contributes to the ongoing advancement of rapid, cost‐effective, and accessible diagnostic tools for early pathogen identification and control of multidrug‐resistant infections.

## 2 Materials and Methods

### 2.1 Bacterial Strains and Culture Media

The bacterial strains used in this study included *Klebsiella pneumoniae* ATCC 9997 as the target strain and five non‐target bacterial strains—*K. pneumoniae* ATCC 700603, *Bacillus cereus* ATCC 11778, *Pseudomonas aeruginosa* ATCC 27853, *Escherichia coli* ATCC 25922, and *Pasteurella aerogenes* ATCC 27783—as negative controls. All bacterial strains were cultured in nutrient broth at 37°C.

### 2.2 Genomic DNA Extraction

Genomic DNA was extracted from bacterial cultures grown in liquid medium at the exponential growth phase. Cells were harvested by centrifugation at 14,000 rpm for 1 min and the pellets were resuspended in 1 mL of phosphate‐buffered saline (PBS). This washing step was repeated twice. The final pellets were resuspended in 20 µL of molecular biology‐grade water and boiled at 98°C for 10 min in a water bath to lyse the cells. The lysates were centrifuged at 14,000 rpm for 5 min, and the supernatants containing crude DNA were collected and stored at −20°C until use.

### 2.3 Oligonucleotide Primers Design

Primers specific to the *K. pneumoniae* virulence genes K2A and orf10 were designed using the MP Primer software, based on sequences obtained from GenBank (accession numbers EF221827.1 and AB362367.1, respectively; Table 1). Primer quality was evaluated using the Integrated DNA Technologies (IDT) online tool (http://www.idtdna.com). Primer specificity was confirmed through BLAST analysis at the National Center for Biotechnology Information (NCBI). The sequence 5′‐CAACCATGGTGGTCGATTAG‐3′ from the *K2A* gene was selected as the probe for the colorimetric assay.

**Table 1:** Primers used for duplex PCR.

Target genes	DNA sequence	NCBI accession no.	Amplicon size (bp)
*K2A*	Forward‐5'‐CAACCATGGTGGTCGATTAG‐3' Reverse‐5'‐TGGTAGCCATATCCCTTTGG‐3'	EF221827.1	532
*orf10*	Forward‐5'‐ACTAGCCTGCAAAGTATCGGGGG‐3' Reverse‐5'‐TAAGGCGGCGCCAGCTCGAATA‐3'	AB362367.1	309

### 2.4 Duplex PCR of *Klebsiella pneumoniae* Serotype K2

All PCR reactions were performed using a thermal cycler (Eppendorf, Germany) in a final reaction volume of 15 µL. The PCR mixture contained 8 µL of 2× Taq Master Mix (Tris‐HCl, pH 8.5; (NH₄)₂SO₄; 2 mM MgCl₂; 0.4 mM dNTPs; 0.2 U/µL Ampliqon Taq DNA polymerase), 10 pmol of each forward and reverse primer, and approximately 100 ng of extracted genomic DNA as template.

Thermal cycling conditions were as follows: an initial denaturation at 94°C for 4 min; 30 cycles of denaturation at 94°C for 40 s, annealing at gradient temperatures of 50.8°C, 54.1°C, 56.7°C, 58.8°C, and 59.9°C for 30 s, and extension at 72°C for 40 s; followed by a final extension at 72°C for 5 min.

PCR products were analyzed by electrophoresis on 1% agarose gels run at a constant voltage of 80 V for 1 h. Gels were stained with ethidium bromide (2 µg/mL) for 10 min and visualized under UV illumination using a UV Doc imaging system (UviTec, Iran).

#### 2.4.1 Determination of Duplex PCR Specificity and Sensitivity

To evaluate the specificity of the duplex PCR assay, five non‐target bacterial strains were tested: *Klebsiella pneumoniae* ATCC 700603 (serotype K6), *Bacillus cereus* ATCC 11778, *Pseudomonas aeruginosa* ATCC 27853, *Escherichia coli* ATCC 25922, and *Pasteurella aerogenes* ATCC 27783.

For sensitivity analysis, serial dilutions of *K. pneumoniae* serotype K2 genomic DNA were prepared at concentrations ranging from 0.0005 to 25 ng/µL. Amplification results were evaluated to determine the lowest detectable DNA concentration.

### 2.5 Synthesis and Characterization of AuNPs

Gold nanoparticles (AuNPs) were synthesized via citrate reduction of HAuCl₄, as previously described (Turkevich et al. 1951). Briefly, 95 mL of an aqueous solution containing 5 mg of HAuCl₄ (Sigma‐Aldrich, USA) was heated to 70°C, followed by the addition of 5 mL of 1% (w/v) sodium citrate solution (Na₃C₆H₅O₇, Sigma‐Aldrich, USA). The mixture was heated and stirred continuously until the color changed from yellow to red, indicating AuNP formation. The resulting colloidal solution was stored at room temperature under dark conditions. The morphology and dispersion of the AuNPs were characterized by UV–Vis spectroscopy (UV2100, Germany) and transmission electron microscopy (TEM).

### 2.6 Colorimetric Assay

Detection of *K. pneumoniae* serotype K2 DNA was carried out using an unmodified AuNP‐based colorimetric assay. In this method, the extracted target DNA was incubated with 15 µL of 15 pM probe in 10 µL phosphate buffer (0.01 M phosphate buffer containing 0.25 M NaCl solution, pH 7.4). The final concentration of NaCl was adjusted to 0.1 M. The mixture was heated at 95°C for 5 min and then hybridized at 55°C for 30 min. After cooling to room temperature, 120 µL of AuNP solution (1 mM) was added to the reaction tube. The color change from red to purple was immediately monitored using a UV–Vis spectrophotometer within the wavelength range of 400–700 nm.

#### 2.6.1 Determination of Colorimetric Assay Specificity and Sensitivity

The specificity of the colorimetric assay was evaluated using non‐target bacterial strains, including *K. pneumoniae* ATCC 700603 (serotype K6), *Bacillus cereus* ATCC 11778, *Pseudomonas aeruginosa* ATCC 27853, *Escherichia coli* ATCC 25922, and *Pasteurella aerogenes* ATCC 27783.

Sensitivity of the assay was determined by testing serial dilutions of *K. pneumoniae* serotype K2 genomic DNA ranging from 0.001 to 25 ng/µL.

## 3 Results

### 3.1 Duplex PCR of *K. pneumoniae* Serotype K2

**Figure 1:**
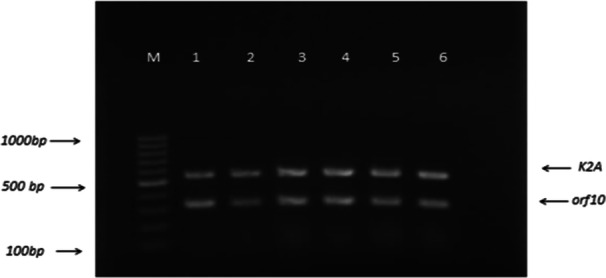
Agarose gel electrophoresis of the gradient duplex PCR products. Length of fragments was 532 bp for *K2A* gene and 309 bp for *orf10* gene. M: 100 bp DNA ladder.1: 50°C, 2: 50.8°C, 3: 54.1°C, 4: 56.7°C, 5: 58.8°C, 6: 59.9°C.

Figure 1 indicates the results of duplex PCR products obtained using gradient PCR. The optimum annealing temperature for duplex PCR was 54.1°C and 56.7°C that showed sharp amplified products. In addition, the duplex PCR results indicated amplification of 532 and 309 bp products corresponded to the *K2A orf10* genes respectively (Figure 1).

#### 3.1.1 The Specificity and Sensitivity of Duplex PCR

**Figure 2:**
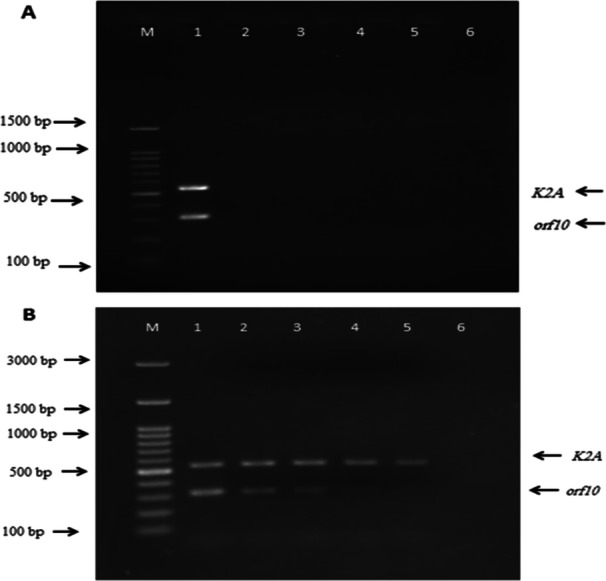
(A) Specificity of the *K2A* and *orf10* primers for detection of. *K. Pnumoniae* serotype K2 by duplex PCR. M: DNA ladder, 1: *K. pnuemoniae* ATCC 9997, 2: *K. pnuemoniae* ATCC 700603, 3: *Bacillus cereus* ATCC 11778, 4: *Pseudomonas aeruginosa* ATCC 27853, 5: *E. coli* ATCC 259226, *Pasteurella aerogenes* ATCC 27783. (B) Sensitivity of the *K2A* and *orf10* primers for detection of *K. Pnumoniae* serotype K2 by duplex PCR. M: DNA ladder, 1: 25 ng/µL, 2: 1.56 ng/µL, 3: 0.07 ng/µL, 4: 0.009 ng/µL, 5: 0.001 ng/µL, 6:0.0005 ng/µL.

Results of duplex PCR using DNA of *K. pnuemoniae* serotype K2 showed amplification of 532 and 309 bp fragments corresponded to the *K2A* and *orf10* genes, respectively. The lack of amplification of the genes in negative control bacteria, suggested the specificity of the designed primers for detection of *K. Pneumoniae* serotype K2 (Figure 2A).

Detection limit for the sensitivity of the duplex PCR assay, using different concentrations of the bacterial genomic DNA showed that the method was able to detect at least 1 pg/µL of the DNA by *K2A* gene and 0.07 ng/µL of the DNA by *orf10* gene (Figure 2B).

### 3.2 Synthesis and Characterization of AuNPs

**Figure 3:**
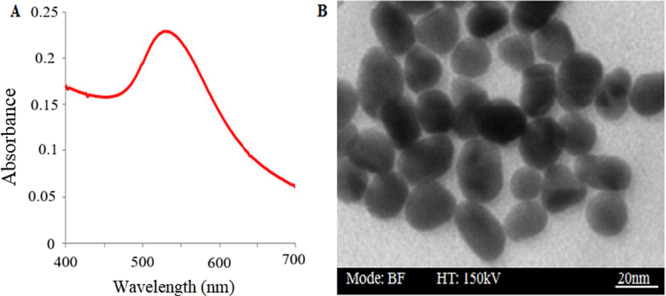
Verification of AuNPs synthesis; (A) UV‐vis spectra from 400 to 700 nm; B) TEM electron microscope image.

According to Figure 3, the results of the TEM indicated formation of spherical AuNPs without aggregation at 20 nm diameter. Also, the solution of gold nanoparticles demonstrated absorbance at 528 nm.

### 3.3 Colorimetric Assay for Detection of *K. pnuemoniae* Serotype K2

**Figure 4:**
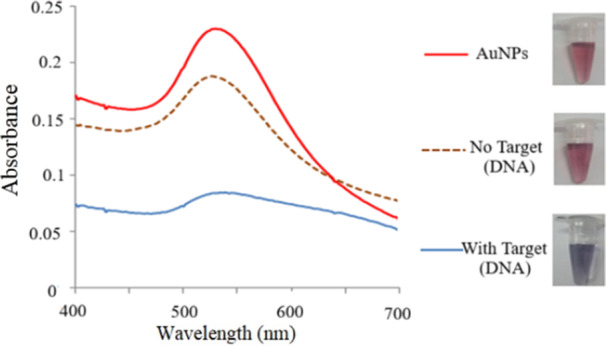
Change in color and absorbance spectrum of unmodified AuNPs before and after hybridization of the DNA probe with genomic DNA of *K. pneumoniae* serotype K2.

The technique involved spectrophotometric and optical evaluation of the solutions before and after AuNPs accumulation. Figure 4 shows the color change and absorbance spectrum of gold nanoparticles solution in the presence and absence of the *K. pnuemoniae* genomic DNA. When the target DNA is not in the environment, the single‐stranded probes attach to the AuNPs. As a result, no changes in color and absorbance spectrum of the solution would be observed.

In the presence of the genomic DNA, after hybridization of the probe with target DNA, the absorbance spectra of AuNPs solution changed from 528 to 550–650 nm and color changed from red to purple (Figure 4).

#### 3.3.1 Determination of Colorimetric Assay Sensitivity and Specificity

**Figure 5:**
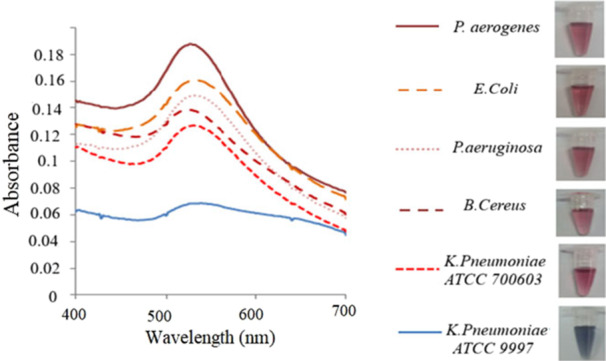
Determination of the DNA probe specificity in colorimetric detection using extracted DNA of 1: *K. pnuemoniae* ATCC 9997, 2: *K. pnuemoniae* ATCC 700603, 3: *Bacillus cereus* ATCC11778, 4: *Pseudomonas aeruginosa* ATCC 27853, 5: *E. coli* ATCC 25922, 6: *Pasteurella aerogenes* ATCC 27783 as negative controls.

**Figure 6:**
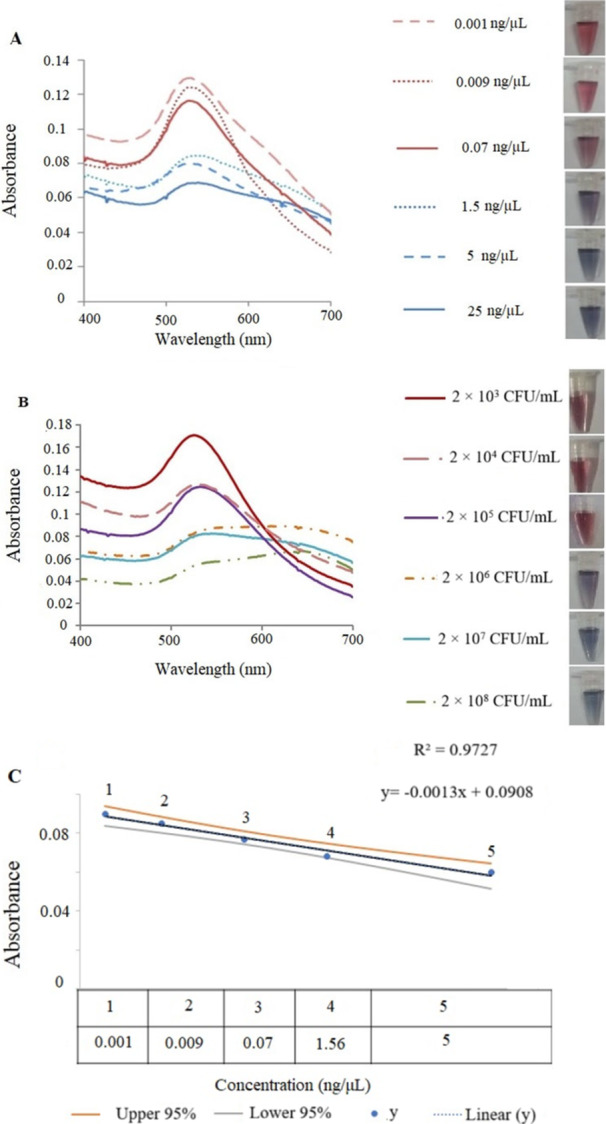
(A) Determination of the DNA probe sensitivity in colorimetric assay in the presence of different concentrations of bacterial genomic DNA: 1: 25 ng/µL, 2: 5 ng/µL, 3: 1.56 ng/µL, 4: 0.07 ng/µL, 5: 0.009 ng/µL, 6: 0.001 ng/µL. (B) Sensitivity determination of the DNA probes in the presence of different CFUs: 2 × 10^8^ CFU/mL; 2 × 10^7^ CFU/mL; 2 × 10^6^ CFU/mL; 2 × 10^5^ CFU/mL; 2 × 10^4^ CFU/mL and 2 × 10^3^ CFU/mL. The minimum concentration of the target DNA caused a change in color and wavelength was 2 × 10^6^ CFU/mL. (C) The linear regression curve displays the relationship between different concentrations of the bacterial genomic DNA and absorbance. (D) The regression line with 95% confidence interval shows a strong correlation (*R*² = 0.9727), confirming the linearity of the response.”

To determine the specificity of the probe in detection of *Klebsiella pneumonia* ATCC 700603, other bacteria such as *Bacillus cereus* ATCC 11778, *Pseudomonas aeruginosa* ATCC 27853, *Escherichia coli* ATCC 25922, and *Pasteurella aerogenes* ATCC 27783 as negative controls were used. Due to the absence of *K2A* gene in negative control bacteria, changes in color and absorption spectrum were not observed (Figure 5). Sensitivity of the primer in the colorimetric detection was determined using different genomic DNA concentrations of extracted DNA from *K. pnuemoniae* and results showed the changes in color and spectrum of solution in concentrations of 25 to 1.56 ng/µL. In the concentration of 0.07 ng/µL, although changes in color were observed, there was no change achieved for the spectrum. Therefore, the least concentration of the genomic DNA required for detection of *K. pnuemoniae* serotype K2 by colorimetric assay was 1.56 ng/µL (Figure 6A). The sensitivity of the DNA probes was determined at concentrations of 2 × 10^8^ to 2× 10^3^ CFU/mL. At 2 × 10^6^ CFU/mL genomic DNA dilution change in wavelength with a slight change in color was observed (Figure 6B). Additionally, a linear relationship (*R* ^2^ = 0.9727) was observed between absorbance and various concentrations of the bacterial genomic DNA. The existence of this linear relationship between the absorption and concentration of bacterial genomic DNA, expressed the accuracy of the reaction sensitivity (Figure 6C). To ensure the reliability of the results, all data points were assessed within the 95% confidence interval of the regression line. Despite minor visual deviations of some points, the high correlation coefficient (*R*² = 0.9727) and the inclusion of all points within the confidence interval confirm that the relationship can be accurately described as linear over the tested concentration range (Figure 6C).

## 4 Discussion

*Klebsiella pneumoniae* serotype K2 is one of the most virulent and clinically significant capsular types associated with urinary tract infections, bacteremia, pneumonia, and liver or brain abscesses. The *K2A* gene encodes a capsular polysaccharide biosynthesis protein that serves as a specific genetic marker for serotype K2, while the *orf10* gene is essential for capsule formation and bacterial pathogenicity. Traditional diagnostic approaches such as biochemical tests, Quellung reaction, and immunoelectrophoresis have limited diagnostic value due to low sensitivity, time consumption, and the high cost of antisera preparation (Gierczyński et al. 2007; Adzitey et al. 2013; Mansour 2014). Consequently, rapid and reliable molecular assays are critical for the identification of *K. pneumoniae* serotypes, particularly hypervirulent strains.

Duplex PCR has been widely adopted as a rapid and reliable molecular approach for simultaneous amplification of multiple targets. Studies have demonstrated that *K2A* and *orf10* genes provide highly specific identification of K2 serotypes in isolates from liver abscesses and other clinical samples (Al‐Jailawi et al. 2014; Doud et al. 2009; Bialek‐Davenet et al. 2014). In the current study, the designed duplex PCR successfully amplified *K2A* (532 bp) and *orf10* (309 bp) fragments with a detection limit of 1 pg/μL and 0.07 ng/μL, respectively. These results indicate that our duplex PCR system provides high specificity and sensitivity comparable or superior to previously reported methods. Besides, EXORCA (EXtraction‐free One‐pot RPA‐CRISPR/Cas12a assay) allow rapid, highly sensitive, and extraction‐free detection of *Klebsiella pneumoniae* from clinical samples. Combined with aptamer‐based colorimetric sensors, these approaches offer fast, reliable, and user‐friendly UTI diagnostics. Fu and colleagues developed the EXORCA assay, which can be completed in approximately 30 min at a constant temperature and allows results to be visualized either with a fluorescence reader or directly by the naked eye under blue light. Evaluation with 20 unextracted clinical samples showed 100% positive and negative predictive values compared to qPCR, highlighting its potential as a rapid, point‐of‐care diagnostic tool for Klebsiella pneumoniae (Fu et al. 2025).

While PCR remains a gold‐standard molecular technique, it requires thermocyclers, electrophoresis, and skilled personnel, which limit its routine application in resource‐limited settings (Wen and Zhang 2012). In this context, nanobiosensors have emerged as transformative tools for medical diagnostics due to their simplicity, rapid response, and high accuracy (Rabiee et al. 2019; Osmani Bojd et al. 2017; Ghazi et al. 2018; Zhou et al. 2022; Najafabad et al. 2022). Among nanomaterials, gold nanoparticles (AuNPs) are preferred for their optical stability, biocompatibility, and strong surface plasmon resonance properties, enabling visible color changes upon nucleic acid hybridization.

Colorimetric assays using AuNPs are generally categorized into crosslinking and non‐crosslinking approaches. The non‐crosslinking method, used in this study, is faster and more straightforward because it does not require probe modification or complex conjugation steps.

Single‐stranded DNA (ssDNA) adsorbs onto the surface of AuNPs via non‐covalent adsorption, forming a protective layer that prevents nanoparticle aggregation. Upon addition of the complementary target DNA, the surface‐bound probe hybridizes with the target, forming a double‐stranded DNA (dsDNA) structure. The dsDNA binds less strongly to the AuNP surface; therefore, when NaCl is added, the nanoparticles aggregate, resulting in a visible color change of the solution (red → blue/purple). Finally, in this assay, the solution color, intensity, and SPR peak shift serve as a specific signal for the target DNA (Wang et al. 2016).

Recent studies have confirmed the effectiveness of unmodified AuNPs in pathogen detection. For instance, Askaravi et al. (2017) applied unmodified AuNPs with peptide nucleic acids for detecting bovine viral diarrhea virus RNA, and Shokri et al. (2017) reported a detection limit of 5 × 10⁻⁹ M for citrus tristeza virus DNA using a similar approach. Ghazi et al. (2018) also demonstrated the feasibility of unmodified AuNPs for detecting gene expression with high specificity.

Recent advances further support the clinical potential of nanodiagnostics. Celik et al. (2023) and Parkhe and Tiwari (2024) emphasized the versatility of AuNPs and hybrid nanomaterials for sensitive, rapid pathogen detection through visible readouts, while Mousivand et al. (2023) highlighted their applicability for detecting bacterial DNA without amplification. Moreover, Mayaka and Alocilja (2025) developed an AuNP‐based biosensor capable of distinguishing multidrug‐resistant bacteria within 45 min, confirming the feasibility of colorimetric detection in clinical diagnostics. In another study, Deb and co‐workers reported a plasmonic gold nanoparticle–based colorimetric aptasensor for the rapid and selective detection of *Klebsiella pneumoniae* in UTI‐related samples with a LoD as low as 3.4 × 10^3^ CFU/mL. The assay relied on the specific interaction between the aptamer and the target bacterium, leading to a pronounced SPR shift and visible color change, while non‐target bacteria produced negligible responses (Deb et al. 2023). This technique is not limited to clinical pathogens and can also be applied to plant pathogenic bacteria such as Burkholderia cepacia, the causative agent of onion sour rot. These findings demonstrate the versatility of gold nanoparticle–based colorimetric detection across different fields. Khoshraftar et al. (2024) developed a AuNPs–based colorimetric assay for the detection of *Burkholderia cepacia*, the causative agent of onion sour rot. Using gene‐specific probes, the method showed 100% specificity and achieved a detection limit of 74.4 × 10⁻⁸ ng/µL of genomic DNA, which was markedly more sensitive than multiplex PCR. This study demonstrates the applicability of gold nanoparticle‐based colorimetric platforms beyond clinical bacterial diagnostics (Khoshraftar et al. 2024).

Our study builds upon this progress by introducing an unmodified, thiol‐free AuNP‐based colorimetric assay targeting the *K2A* gene of *K. pneumoniae* K2. Compared to our previous work using thiolated probes, this simplified approach eliminates surface modification, significantly reduces cost, and shortens detection time. The assay displayed high specificity and a sensitivity limit of 1.56 ng/μL, producing a clear color change in under 60 min. Importantly, the method required only extracted DNA, with no need for PCR amplification, labeling, or electrophoresis, demonstrating that *direct DNA‐AuNP interaction* is sufficient for reliable identification.

Together, the duplex PCR and unmodified AuNP colorimetric methods form a complementary diagnostic strategy. Duplex PCR provides confirmatory, highly sensitive molecular identification, while the AuNP‐based colorimetric assay offers a rapid, cost‐effective, and instrument‐free detection platform suitable for point‐of‐care applications. The findings align with recent trends in molecular biotechnology emphasizing field‐deployable, low‐cost nanodiagnostic platforms for pathogen detection (Najafabad et al. 2022; Wu et al. 2021).

## 5 Conclusions

In this study, two efficient diagnostic assays were developed and evaluated for the detection of *Klebsiella pneumoniae* serotype K2 using the *K2A* and *orf10* genes. The duplex PCR method demonstrated high specificity and sensitivity, confirming its reliability for the identification of *K. pneumoniae* in both clinical and environmental samples.

More notably, the unmodified AuNP‐based colorimetric assay using a 20 bp *K2A* probe provided a rapid, label‐free, and cost‐effective diagnostic alternative, eliminating the need for modified probes, amplification, or sophisticated instruments. The entire detection process was completed in less than 60 min, with a clear visible color shift, supporting its potential use for point‐of‐care and low‐resource laboratory diagnostics.

These findings underscore the practical advantages of thiol‐free AuNP‐based detection and demonstrate that simple, direct DNA‐AuNP interactions can offer sensitive, specific, and rapid bacterial identification. This approach represents a promising diagnostic alternative for early detection and effective control of *K. pneumoniae* infections, consistent with emerging trends in nanodiagnostic technology (Celik et al. 2023; Parkhe and Tiwari 2024; Mayaka and Alocilja 2025).

## Author Contributions

**Sepideh Ahmadi:** writing – original draft, investigation, methodology, data curation, formal analysis. **Fatemeh Haddadi:** conceptualization, funding acquisition, supervision, validation, visualization, writing – review and editing, project administration. **Hossein Kamaladini:** conceptualization, supervision, project administration, visualization, writing – review and editing.

## Ethics Statement

The authors have nothing to report.

## Conflicts of Interest

The authors declare no conflicts of interest.
